# An evaluation of the Victorian Salt Reduction Partnership’s advocacy strategy for policy change

**DOI:** 10.1186/s12961-021-00759-1

**Published:** 2021-07-15

**Authors:** Emalie Rosewarne, Michael Moore, Wai-Kwan Chislett, Alexandra Jones, Kathy Trieu, Jacqui Webster

**Affiliations:** The George Institute for Global Health, The University of New South Wales, Sydney, NSW 2006 Australia

**Keywords:** Public health advocacy, Public health policy, Salt reduction, Population intervention

## Abstract

**Background:**

Public health advocacy strategies facilitate policy change by bringing key health issues to the forefront of public and political discourse, influencing decision-makers and public opinion, and increasing policy demand. The Victorian Salt Reduction Partnership (VSRP) was established in 2014 in response to inadequate government action to improve population diets in Australia. This study aimed to evaluate the success of the VSRP’s advocacy strategy in achieving policy change.

**Methods:**

Documentation of VSRP activities and outputs were collected, and semi-structured interviews conducted as part of a comprehensive process evaluation. For this study, the Kotter Plus 10-step public health advocacy evaluation framework was used to guide data extraction, analysis, and synthesis.

**Results:**

A sense of urgency for salt reduction was generated by producing evidence and outlining the potential impact of a state-based salt reduction programme. This enabled the creation of a coalition with diverse skills and expertise, which facilitated the development of an innovative and collaborative advocacy action plan. A clear change vision was established, but communication of the vision to decision-makers was lacking, which reduced the impact of the programme as decision-makers were not provided with a clear incentive for policy change. As a result, while programme outputs were achieved, these did not translate to achieving broader strategic goals during a limited-term intervention in a political climate unconcerned with salt.

**Conclusions:**

The Kotter Plus 10-step framework was a useful tool for evaluating the success of the VSRP advocacy strategy. The framework enabled the identification of key strengths, including the creation of the guiding coalition, and areas where efforts could be improved in future similar strategies, such as effective communication within partnerships and to decision-makers, to better influence policy and improve public health impact.

## Background

Governments are often reluctant to introduce preventative policies and initiatives to reduce risk factors for noncommunicable diseases (NCDs), such as poor diets [1, 2]. Reasons for this include lack of public support and political will [1]. Across public health disciplines, it has been consistently shown that scientific evidence alone is not sufficient to influence the public and policy-makers [3, 4]. Public health advocacy strategies bring key health issues to the forefront of public and political discourse and thus increase the demand for policy change [3, 4]. Public health advocacy has been defined by Moore as “the deliberate process of using knowledge and evidence to support or argue in favour of a cause, policy or idea in order to influence decision-makers and public opinion to deliver better population health outcomes” [5]. This involves translating important scientific evidence into easy-to-understand, resonant messages for a general audience; intentional and strategic framing of the public health problem and solution; and persistent efforts to persuade public opinion and decision-makers [4, 6].

In Australia, strategic public health advocacy has contributed to the uptake of effective policies that have saved countless lives in areas such as tobacco control and injury prevention [4, 6, 7]. Although the Australian government has committed to achieving the WHO global NCD targets by 2025, including the diet-related goals of a 30% reduction in population salt intake and stopping the rise in diabetes and obesity [8], there are no coordinated national strategies and policies to improve population diets [9]. In the absence of comprehensive policy action, Australian deaths and disability from diet-related NCDs remain high, with 22,000 deaths and 393,000 disability-adjusted life years attributed to poor diets in 2019 alone [10]. Only piecemeal actions have been taken by the Federal Government towards the global NCD targets. In 2014, a voluntary front-of-pack nutrition labelling system, the Health Star Rating System, was introduced to promote healthier diets [11] by supporting consumers to make informed food choices [9]. Yet, after 5 years, only around 40% of eligible products displayed a Health Star Rating, and the impact on consumer choices was limited as manufacturers mostly used the label on healthier products [12]. In 2020, after almost 5 years of planning, voluntary nutrient reformulation targets for processed foods were released by the Federal Government’s Healthy Food Partnership to improve the food supply by reducing levels of risk-associated nutrients (sodium, saturated fat, and sugar) [13]. While it is too early to evaluate impact, preliminary assessments indicate the targets have not been set for a wide enough range of foods and are too conservative. This means that even if the targets were fully achieved, they will have a limited effect on food composition and population health [14, 15].

In response to inadequate Federal Government action to improve population diets, the Victorian Health Promotion Foundation (VicHealth) established the Victorian Salt Reduction Partnership (VSRP) in 2014. The VSRP has since worked to implement a multifaceted intervention to reduce population salt intake in the state of Victoria by 1 g by 2020 [16]. A key component of the VSRP intervention was advocacy for salt reduction policy change at state and federal levels of government, which was supported by activities to engage the food industry and generate public debate to strengthen support for the advocacy agenda.

**Fig. 1:**
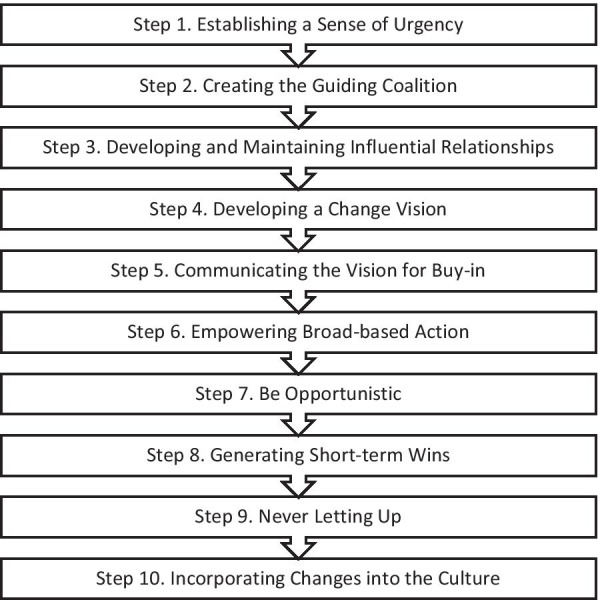
Moore, Yeatman, and Pollard’s “Kotter Plus—a 10 Step Plan” [17]

This study aimed to evaluate the success of the VSRP’s advocacy strategy for policy change using Moore, Yeatman, and Pollard’s [17] 10-step public health advocacy evaluation framework (Fig. 1). This framework was built on Kotter’s eight-step change management process and has been adapted for public health [18]. The purpose of using this framework was to evaluate the VSRP advocacy strategy by developing an understanding of the policy change process and factors influencing this, including why and how advocacy activities did or did not lead to policy change. This research is part of a comprehensive process evaluation of the VSRP’s intervention strategy [19].

## Methods

### Data sources and collection

Detailed methods for data collection for the process evaluation are published elsewhere [19, 20]. Briefly, documentation of activities and outputs were regularly collected throughout the implementation period as part of the comprehensive process evaluation [19]. These data were collated for each intervention component and entered into an Excel spreadsheet that was purpose-built for the process evaluation [19].

Semi-structured interviews were undertaken to obtain stakeholder perspectives on the effectiveness of the VSRP following the intervention [20]. Interviewees comprised 14 VSRP stakeholders [four from research organizations, six from nongovernmental organizations (NGO), four from State Government or statutory agencies (SGSA)] and seven food industry stakeholders [20]. Interviews were transcribed, imported into NVivo for data management, and had been previously thematically analysed using the Consolidated Framework for Implementation Research (CFIR) to understand the effectiveness of the VSRP and factors affecting programme implementation [20, 21].

### Data extraction and analysis

For this evaluation, Moore, Yeatman, and Pollard’s public health advocacy evaluation framework [17] was used to guide data extraction, synthesis, and secondary analysis of the process evaluation and interview data. As this framework can be used to both plan and evaluate public health advocacy strategies [17], it was then used to evaluate the success of the VSRP’s advocacy strategy, including whether key elements of successful advocacy were present and areas for improvement for future advocacy efforts.

### Ethics and consent

This study was approved by the University of Sydney Human Ethics Research Committee (2016/770). For the interviews, written informed consent was obtained from all participants before the interview.

## Results and discussion

### Step 1. Establishing a sense of urgency

A sense of urgency is established when others see the need for policy change, are convinced of the importance of the public health issue, and are prepared to take immediate action [18]. The policy argument for reducing salt intake at a population level stems from the health risks associated with salt overconsumption; namely, increased blood pressure and increased risk of cardiovascular diseases and other NCDs [22]. In 2012, mean salt consumption in Victoria was estimated to be 8.6 g per day for men and 6.3 g per day for women [23], well above the recommended daily maximum of 5 g per day [24]. The VSRP undertook the following steps to establish a sense of urgency: (1) consolidating the evidence base for salt reduction interventions and making context-specific recommendations, (2) estimating the potential Victorian lives that could be saved and healthcare costs averted if salt reduction was achieved, and (3) identifying current government action in relation to the global targets.

Specifically, in 2013, VicHealth commissioned The George Institute for Global Health to develop the evidence base around population-level salt reduction and create the case for action in Victoria. Three scoping activities were undertaken:

1. Reviews of state- and community-level salt reduction initiatives, and behaviour change interventions, which determined their effectiveness in reducing population salt intake and established the feasibility of a state-led salt reduction intervention [25, 26].
2. Development of the economic business case for salt reduction in Victoria, which included estimating the potential Victorian lives saved (787 lives per year) and healthcare costs averted ($47 million per year) from a 3 g reduction in average population salt intake [27].
3. Assessment of existing salt reduction activities in Australia to identify population needs and current gaps in government action towards the global target [28].

This evidence was used to bring together key experts on salt to create the VSRP (step 2) and inform the development of the VSRP strategy and joint action plan (step 4).

Whilst VSRP members were aware of the importance of reducing salt, urgency for salt reduction efforts was not established among consumers, food industry, and policy-makers [29]. Based on the assessment of existing initiatives [28], and reinforced in the stakeholder interviews, it was evident that there was “a lack of recognition of salt as a population health priority in the Australian context” (SGSA 13). Interviewees commonly identified that competing nutrition initiatives, such as sugar reduction and obesity prevention, were more top of mind. In their work on effective advocacy, Cullerton et al. suggest such challenges can be overcome by amplifying messaging to bring public health issues to the forefront of the public domain lack. To this effect, the VSRP used recommended public health advocacy strategies such as media advocacy [30] (including use of personal testimonies [31]), policy champions, and policy position statements to generate public debate, engage the food industry, and draw the attention of policy-makers (steps 5 and 6). However, some interviewees thought that the VSRP could not overcome the noise of competing issues as the its messaging was not strong enough. However, Allen and Feigl suggest the public and policy-makers’ inattention to addressing NCD risk factors is more likely related to how the issue is framed [32]. They propose that attempts made to develop a sense of urgency to address risk factors for the “world’s largest killer” are undermined by the language used [32]. For example, the term “noncommunicable disease” identifies a group of diseases only by what they are not [32] making it difficult to advocate for risk factor prevention strategies. Whether due to a competitive nutrition space or an inability to overcome public and policy-makers’ indifference to NCD prevention, our findings suggest the VSRP did not establish sufficient urgency to optimize its effectiveness. More work and innovative ideas are needed to generate the consumer, industry, and political push needed to drive salt reduction up the policy agenda in Australia.

### Step 2. Creating the guiding coalition

Coalitions have the ability to generate the advocacy needed to create public health policy change, as they serve to amplify the collective voice of the group [33, 34] and allow the pooling of resources, knowledge, and skills [35]. Aligned with evidence of effective partnerships, to create this guiding coalition, VicHealth invited government, nongovernmental, and academic organizations [36] with an interest in salt reduction [35, 37] and a diverse range of skills and expertise [38, 39] to join a strategic partnership that would coordinate and deliver a multifaceted salt reduction initiative in Victoria [40]. Ten organizations formally joined the VicHealth-led coalition: The George Institute for Global Health, Heart Foundation, Deakin University Institute for Physical Activity and Nutrition, Baker Heart and Diabetes Institute, Kidney Health Australia, Stroke Foundation, High Blood Pressure Research Council, Commonwealth Scientific and Industrial Research Organisation, Food Innovation Australia Limited, and the Victorian Department of Health and Human Services (observer only). The health-promotion objectives of partner organizations spanned from primary prevention to tertiary prevention of one or more diseases related to high salt intakes. Once the coalition was established, the VSRP could then develop the programme change vision (step 4). The VSRP met quarterly throughout the intervention period to provide strategic input into the intervention [20].

The food industry was not invited to join the VSRP, so that public health objectives could be set by the VSRP independent of any conflicts of interest. The food industry was then engaged in partnership activities after the change vision (step 4) was established, to accelerate effective salt reduction action in Victoria through collaboration [41].

While the creation of the VSRP fulfilled the role of a guiding coalition, interviews with members highlighted the nuances of working within a diverse partnership toward a shared policy goal. This diversity was viewed as a key strength, which cultivated a positive learning climate [35] and facilitated the development of innovative intervention approaches [42]. However, it may have contributed to communication challenges around strategic priorities [42], such as between members operating at the strategic level and programme implementers who had different views on VSRP goals based on their previous experiences, which may have hindered the effectiveness of the VSRP [37, 43]. Further, 10 of 14 VSRP stakeholders identified compatibility challenges, whereby organizational and individual priorities were in conflict with the collaborative goals [21]. Specifically, during the implementation period, some organizations shifted away from core partnership values, including prioritizing salt reduction, to a focus on general healthy eating principles. In partnerships, this shift often creates tension within the group and uncertainty regarding the strategic direction [44, 45], but “trying to find where we all come together” (NGO 7) and focusing on common goals and values can help prevent this.

Our analysis suggests the creation of a diverse, skilled guiding coalition can facilitate public health advocacy efforts. However, misalignment of priorities and goals may have hindered the effectiveness of the coalition in executing joint advocacy efforts. The risk of this occurring should be considered when approaching potential partner organizations, taken into account in the management of the coalition, and mitigated throughout implementation.

### Step 3. Develop and maintain influential relationships

Developing and maintaining influential relationships with decision-makers is crucial to achieving policy change [29]. The VSRP was able to do this by (1) inviting individuals and organizations with an interest in salt reduction who already had established relationships with decision-makers to join, (2) raising the profile of the VSRP to establish it as an authority on salt reduction, and (3) building relationships with the food industry to understand competing views and work towards a unified solution.

VSRP members utilized pre-existing relationships with individuals, organizations, and external groups to expand the supporter base for salt reduction and pursue advocacy aims. Interviewees identified enabling relationships, including connections to state and Federal Governments (ministers, minister’s advisors, members of parliament, senators), government departments (e.g. Business Victoria, Department of Jobs, Precincts and Regions Victoria, Australian Government Department of Health), Federal Government initiatives (e.g. Healthy Food Partnership), and food industry (Australian Food and Grocery Council, food manufacturers). While pre-existing relationships were leveraged for strategic advocacy, some stakeholders suggested the VSRP could have done more to better understand the current political climate and policy opportunities within it [46], and proactively engage influential people outside of pre-existing relationships, which likely would have facilitated progress towards policy change.

To establish the VSRP as an authority on salt reduction in Australia [29], partner organizations raised its profile through communication with stakeholder networks, events and activities, presentations at conferences, reports, and peer-reviewed publications. Some interviewees discussed the success of VSRP gaining credibility in the nutrition space and becoming an entity that some food manufacturers approached for support [29]. Another example of its success was when the Healthy Food Partnership disseminated VSRP resources to the food industry to support companies to meet the nutrient reformulation targets (steps 5 and 6) [47].

Finally, the VSRP aimed to build relationships with the food industry, to understand industry stakeholders’ points of view on, and provide support for, food reformulation to reduce salt. Previous research has demonstrated that the food industry in Australia has stronger networks, and more influence over, political decision-makers than nutrition coalitions [29]. By building positive relationships with food industry stakeholders, which facilitated knowledge exchange and enabled a deeper understanding of capabilities and challenges [20], the VSRP could work with food industry towards a mutual goal of salt reduction in packaged foods [29]. In overcoming the “us and them mentality” (Industry 16) between public health and food industry and working together, the VSRP and industry stakeholders could exert joint influence on decision-makers and other food companies.

Individuals join coalitions with pre-existing relationships with influential people and organizations, which can be leveraged to expand the supporter base for public health causes. However, our analysis indicated coalitions need to build and maintain new influential relationships with a range of stakeholders, including the food industry, and work to raise the profile of the coalition to have the best chance of success.

### Step 4. Develop a change vision

**Fig. 2:**
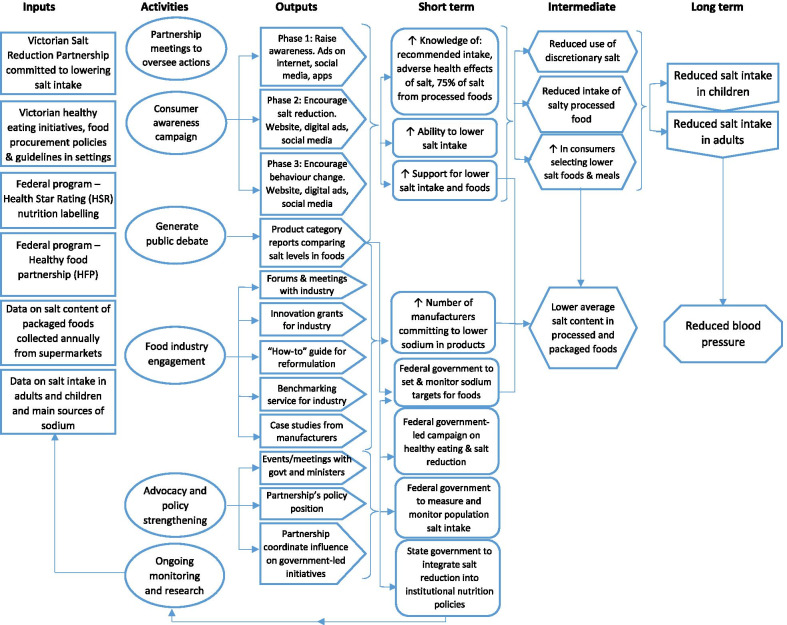
Revised logic model of the Partnership program. Adapted by Rosewarne et al. [20] from Trieu et al. [19]

The overall VSRP change vision is illustrated in the programme logic model [19, 20] (Fig. 2). It was established through an intervention strategy and joint action plan [40], which were informed by the formative VicHealth-commissioned research [25–28]. Briefly, five key actions areas were determined: building strong partnerships, generating public debate, increasing consumer awareness, advocacy and policy strengthening, and food industry engagement. Public-facing documents and materials outlining the strategy were developed [27, 40, 48], which “summarised the health and economic case for salt reduction” [49] in Victoria and built on the previously established sense of urgency [25–28].

The policy change vision was for state and Federal Governments to establish policies to create a healthier food supply including salt reduction strategies [40]. Specific policy goals were for the Federal Government to set and monitor targets to reduce salt in identified food categories, measure and monitor changes in population salt intake, and deliver a national healthy eating campaign (including a focus on the importance of reducing salt consumption); and for the Victorian State Government to integrate salt reduction into institutional nutrition policies (Fig. 2, short-term outcomes).

To achieve these policy objectives, an advocacy and policy-strengthening agenda was developed, which was supported by strategies to engage the food industry and generate public debate through media activities (Fig. 2, activities). Through accomplishing specific programme outputs, the intention was that these strategies would increase public and political awareness and push for salt reduction (Fig. 2, outputs).

Interviewees shared their perspectives about the advocacy agenda, and it was evident that there was a lack of clarity around the overall policy change vision [39]. One stakeholder thought the VSRP “just added a stronger supporter base around things that were already in train” (SGSA 11), and another suggested the VSRP had “certainly affected things that have been going on” (NGO 21), such as the Federal Government setting salt targets for foods. However, other interviewees held a different perspective, stating there were “really clear efforts” (Research 1) to advocate for policy change through a variety of means such as a policy position statement, meetings and events with government and decision-makers, and letters to ministers and other policy stakeholders (steps 5 and 6).

The interviews highlighted that many were unaware of the full extent of the advocacy agenda. Only two of 14 VSRP stakeholders shared that there were Victorian State Government-based objectives in addition to Federal Government goals, and they felt very little progress was made towards these. Inconsistencies in members’ understanding of the action area aims and objectives influenced how individuals felt about the change vision overall and likely impacted the execution of VSRP activities [36, 39]. Disconnect between strategic goals and individual or organizational perceptions of these can ultimately result in miscommunication and/or evolution of goals and lead to a drift away from the change vision [45]. Evidence from the stakeholder interviews suggests implementation team members, who were contracted to deliver the intervention within the programme time frame, were focused on fulfilling contractual obligations rather than striving to achieve the overall change vision. Over time, due to individual key performance indicators and staff turnover, this resulted in gaps in activities contributing to strategic goals. For effective outcomes, partnerships should make a deliberate effort to prevent evolution of goals and drift from the change vision by orienting all individual, organizational, and group activities within the framework of the change vision and ensuring all members have a strong understanding of the coalition’s strategic goals.

### Steps 5 and 6. Communicate the vision for buy-in and empower broad-based action

Coalitions can “make sure as many as possible understand and accept the vision and the strategy” [18], by identifying and creating different opportunities to communicate the vision and build relationships with stakeholders to empower broad-based action. To communicate the change vision to policy-makers, and attempt to stimulate government action, three activities were undertaken by the VSRP: (1) development of an advocacy asks document, “Reducing the pressure on our health and economy” [50], (2) parliamentary events and meetings with ministers, and (3) influencing government-led initiatives, such as the Federal Government’s Healthy Food Partnership and Health Star Ratings committees, and State Government’s institutional nutrition policies (e.g. Healthy Choices). In addition, efforts were made to communicate the change vision and empower broad-based salt reduction action within the public and food industry.

The advocacy asks document was developed in consultation with the VSRP members to ensure the change vision was clear and easy to communicate [39]. The document called for the implementation of an effective national food and nutrition strategy, which included salt reduction, and asked the Federal Government to (1) set and monitor targets to reduce salt in identified food categories, (2) measure and monitor changes in population salt intake, and (3) deliver a national healthy eating campaign, including a focus on the importance of reducing salt consumption; as well as outlining steps for measuring success [50]. Many thought that the development of the advocacy asks document was a “milestone”, and gained consensus on a clear change vision for the VSRP, which formed a “blueprint” on what was needed going forward and would be used for joint, coordinated strategic advocacy. Documents such as this, which raise awareness of an issue and clearly outline solutions to the problem, have the potential to have high impact [34]. However, some interviewees shared concerns about ineffective utilization of the document to communicate the change vision to decision-makers due to confusion around roles and responsibilities for document dissemination [51]. This was then a barrier to empowering broad-based action on the three policy asks [17] and hindered the potential of the VSRP to achieve policy change [37, 43]. Relying on passive dissemination to communicate the change vision to decision-makers is less effective than strategic, targeted dissemination [51]. A key lesson was the importance of active, targeted dissemination strategies, with clear allocation of individual and organizational roles and responsibilities, for the greatest chance of reaching and influencing the right decision-makers.

Over the course of the VSRP, three parliamentary events were held (Victorian Parliament: Breakfast, March 2016. Federal Parliament: Lunch, March 2016; Breakfast, August 2018) where State and Federal Ministers, Members of Parliament, and Senators were engaged in conversations about salt reduction. Events and correspondence with policy-makers were an attempt to cement salt reduction as a public health priority, increase the visibility of the VSRP amongst policy-makers and stimulate policy action [34]. Three interviewees shared successes of these activities, such as receiving positive responses from ministers and government officials, and good representation and attendance at parliamentary events [34]. Others felt these communication methods were not enough to “elevate” salt reduction as a priority in the nutrition space and that the strategy had not “quite had the impact at the state and federal level we wanted” (SGSA 10).

In addition to these events, individual VSRP members had opportunities to communicate the change vision through positions on government working groups including the Healthy Food Partnership and Health Star Rating committees. These individuals gained a better understanding of relevant policy-making processes and how the VSRP could influence these processes [34]. The VSRP and partner organizations submitted responses to public consultations (e.g. [52, 53]) to communicate the VSRP vision to these committees. The VSRP also circulated responses to wider stakeholder groups, including public health and consumer groups, to further communicate the VSRP change vision with a view to empowering broad-based action from like-minded organizations.

Policy asks were also communicated to the public and food industry through media advocacy activities. Engaging the media frequently and attracting coverage increases the likelihood of achieving policy change through public demand [34]. Over the intervention period, the VSRP executed nine media advocacy activities highlighting the salt content of different food groups and the VSRP’s change vision [30]. Stakeholders cited these as one of the most “effective” partnership activities for “strategic advocacy”, as they enabled the VSRP to “raise awareness with the public and our policy-makers… to liaise directly with the food companies to raise their awareness… and invite them to the table, and to develop strategies to try and reduce salt” (Research 2). Between 2 and 8 million Australians had the opportunity to see each of these media activities [30]. Whilst this illustrates the potential for gaining public support, there is not yet evidence to suggest increased public support for salt reduction policies [54], which is a key factor for influencing policy-makers [29]. Between one and three manufacturers were met with for each media release, and overall one quarter of manufacturers targeted through these activities were engaged by the VSRP in discussions about salt reduction reformulation and the VSRP change vision [30]. Media advocacy was effective in engaging some food companies, including all four major Australian retailers and three top 100 manufacturers in Australia [30]. However, the effect of these activities in reducing salt levels in the food supply is not yet known. This would be a key driver of further salt reduction action by industry and demonstrate the effectiveness of the approach to policy-makers.

Communicating the change vision and empowering broad-based action amongst the food industry was a key activity in working towards the strategic goal of reducing salt levels in foods through policy change and manufacturer commitment to salt reduction. This was done via: one-to-one meetings, forums and events, innovation grants, benchmarking services, case studies, and a reformulation guide (Fig. 2). The development of the salt reduction reformulation guide [55] provides a clear example of the VSRP communicating the change vision and empowering broad-based action amongst the food industry. Created in collaboration with a food industry expert, the guide outlines nine key steps for product reformulation. Food industry interviewees shared that this was “a great resource” that was “really helpful” for supply chain and food technologists (Industry 17). Some stakeholders suggested it was most useful for small to medium businesses commencing their reformulation journey, while reinforcing the processes larger companies with reformulation experience were already undertaking. Through this resource, manufacturers could be empowered to take the necessary steps to produce lower-salt foods.

The likelihood of achieving broad-based action is increased with wider communication of the vision. The VSRP was profiled as one of the Australian initiatives to achieve United Nations Sustainable Development Goals 3 and 12 [56], allowing communication of the vision to a broad public health audience. Another key achievement of the VSRP was in magnifying its industry engagement strategy through the provision of the reformulation guide to the Federal Government, which then disseminated it to all food manufacturers alongside the announcement of national salt targets [47]. Supplementing this resource, the VSRP held a panel event to launch the guide in May 2019, with more than 60 food industry stakeholders in attendance [57], and an online webinar in July 2019, with more than 50 participants. One food industry interviewee shared that these events were “really beneficial” as they were an opportunity to “have an open discussion and to ask experts questions” (Industry 15). These activities were supported by media and social media activities and served to further communicate the message of the VSRP, and the collaborative and interactive approach allowed a deeper understanding of the VSRP vision and facilitated the food industry’s ability to take action.

Partnerships should plan ways to communicate the change vision and empower broad-based action during the development of a strategy. The VSRP took opportunities to communicate the change vision to policy-makers, government, media, consumers, and industry and attempted to empower broad-based action from each of these stakeholder groups. The process of empowering broad-based action was facilitated by the creation and dissemination of resources that outline solutions to public health problems, such as policy position statements and how-to guides.

### Step 7. Be opportunistic

Successful advocacy relies upon making the most of opportunities as they arise [17]. While many advocacy activities in the VSRP strategy were planned, the ability of the VSRP to adapt to the changing political and decision-making environment allowed it to utilize unanticipated opportunities to fuel its change vision [4].

Overall, interviewees felt that the salt reduction was not a priority in the current political climate and that there were limited opportunities for policy progress. However, many interviewees were pleased with the VSRP’s ability to respond to, and influence, Australia’s proposed salt targets. The process for setting salt reformulation targets commenced with the Federal Government forming the Healthy Food Partnership in 2015 [58], followed by the establishment of the Reformulation Working Group in 2016 [59]. These were both subsequent to the establishment of VSRP and the development of the change vision. Throughout the implementation period, stakeholders perceived the dynamic policy environment, particularly the actions (and inactions) of the Healthy Food Partnership, to influence the VSRP’s advocacy action plan, requiring it to make adaptations to optimize the intervention, while maintaining the overall change vision [60, 61]. An example was the VSRP deciding to respond to the 2018 public consultation on the salt targets, which provided an unplanned opportunity to communicate the change vision and empower broad-based action.

It is important to consider whether responding to new opportunities is aligned with the change vision and whether intervention fidelity, which is crucial to achieving the change vision through the causal pathway established in the logic model, can be maintained [17, 60]. In this example, responding to the public consultation and the change vision were closely aligned. However, it is possible that continually reacting to a changing policy environment over a period of time influenced stakeholders’ perspectives on the overall goals [60, 62]. For instance, some interviewees only spoke about achieving salt targets and overlooked the other advocacy objectives. It is important to ensure opportunities taken to support the broader change vision do not detract attention from the broader strategic goals. A key learning was the use of monitoring and evaluation processes, which included continual data collection for the process evaluation [19] and the midterm evaluation of stakeholder perspectives [63], to ensure intervention fidelity was maintained while also supporting decision-making around adaptations to optimize the intervention.

### Step 8. Generate short-term wins

Public health advocacy is typically long-term work. Generating short-term wins is particularly important in measuring and monitoring advocacy progress [17]. One such way to demonstrate “visible” progress is the generation of specific project outputs, which can then be linked to tangible outcomes.

The VSRP outputs related to the advocacy initiatives were outlined in the initial programme logic model, allowing achievements to be clearly recognized (Fig. 2). In line with this, interviewees felt that planned outputs were generated throughout the duration of the VSRP, including the policy position statement to use for advocacy to government, meetings and events (e.g. parliamentary breakfasts) to engage decision-makers, and shared resources with the Healthy Food Partnership and food industry to empower reformulation action, product category reports to generate public debate, and other industry engagement strategies to support reformulation.

Although project outputs were generated, predicted short-term outcomes were not achieved within the VSRP intervention time frame (Fig. 2). Salt targets were released in May 2020, after the VSRP intervention had ended, reflecting the delay between outputs and outcomes commonly seen in public health interventions. Additionally, generating an output or achieving an outcome may not always result in the overall objective being reached. In this example, whilst the targets were released, our analysis determined the targets were limited in scope and too conservative and will likely have a limited impact on the food supply [14]. Further, a plan for implementation and monitoring has not been created, raising questions about the execution and impact of the initiative. The other two VSRP advocacy asks, a national food and nutrition strategy [64] and a Federal Government programme to measure and monitor population salt intake, have not been established. Finally, the objective of influencing state-based nutrition policies was not achieved, with one stakeholder suggesting “it’s been a challenge to impact on those processes and to know whether or not those policies are being implemented and whether or not they are also incorporating salt in those policies… Because we didn’t have any direct access to those institutional settings as a project” (Research 2).

This means that the VSRP only partially achieved one out of the four stated goals of the change vision, even though project outputs were accomplished. This highlights the importance of conducting a comprehensive process evaluation to understand what was achieved, how it was achieved, and in what context it was achieved.

### Step 9. Never let up

Given that “often it can take twenty or thirty years for the advocacy to reach a critical mass” (SGSA 13) to achieve public health advocacy outcomes, patience and persistence are essential, while waiting for the right leadership, the right resources, the right time, and the right opportunities [65, 66]. Drawing on his experience in tobacco advocacy, Daube summarizes this idea as “overnight success takes time” [65].

The VSRP demonstrated persistence in pursuing its advocacy agenda over the 4-year implementation period despite many challenges. The group built on pre-existing initiatives and sought to strengthen coexisting policies (e.g. Federal Government’s Healthy Food Partnership, Victorian Government’s Healthy Choices) aiming to better integrate salt reduction goals in broader nutrition policies at state and federal levels of government [40]. Many felt that the external policy environment hindered advocacy efforts, specifically the slow progress of the Healthy Food Partnership in setting sodium targets, and one interviewee highlighted challenges in integrating salt reduction objectives into state healthy eating policies. Despite the lack of comprehensive action and slow progress, the VSRP publicly welcomed government actions, such as the development of draft salt targets [67], and shared VSRP resources and lessons with the Healthy Food Partnership.

However, continuity is required [17]. Coalitions are often formed for short-term public health interventions, but it is crucial that organizations continue to be committed to the joint advocacy agenda until policy change is achieved. Toward the end of the VSRP implementation period, there was uncertainty regarding the VSRP’s next steps and whether activities would be sustained. Interviewees suggested it was “critical for the partnership to come together and find how can we keep this momentum where we have momentum” (NGO 7).

Whilst VicHealth is no longer investing in a state salt reduction partnership, there is strong potential for individual organizations working on salt reduction to join forces to lobby on this issue as a coalition at the national level. Continued long-term coalition-based advocacy efforts are necessary to achieve the “critical mass” (SGSA 13) needed to generate further salt reduction action in Australia [6, 33].

### Step 10. Incorporate changes into the culture

To incorporate changes into the political and social culture, the preceding nine steps must be achieved. Missing key elements in this sequence can tarnish the subsequent steps and result in a lack of advocacy outcomes.

In an attempt to change the Australian culture, the VSRP established collaborative approaches with the government and policy-makers, the public, the media and the food industry [16, 30, 40], which allowed the VSRP to work with other stakeholders towards a unified solution to salt reduction [29, 42]. However, in a crowded nutrition space, sufficient public and political drive for salt reduction were not perceived to be generated. Without this, it is unlikely that changes made by the VSRP will be incorporated into the political and social culture.

To summarize, a sense of urgency was generated amongst stakeholders with an interest in salt reduction by producing evidence and outlining the potential impact of a state-based salt reduction programme. This enabled the creation of a diverse and skilled guiding coalition, which facilitated the development of an innovative collaborative advocacy action plan. A clear change vision was established. However, more could have been done to communicate the VSRP vision to, and empower broad-based action amongst, decision-makers, government, the public and the food industry. The fact that this didn’t happen reduced the impact of the advocacy strategy as decision-makers were not provided with a clear incentive for policy change. As a result, only one out of the four stated goals of the change vision was even partially achieved. Unexpected advocacy opportunities were taken, and short-term wins were accomplished, however these did not translate to achieving the overall change vision during a limited-term intervention in apolitical climate where salt is low on the agenda and where decision-makers to continue to prioritize treatment over prevention.

The VSRP intervention demonstrates the complexities of collaborative approaches to advocacy within a multifaceted intervention. The main lessons from the VSRP approach are outlined in Table 1. These lessons add further insights to previous understanding of public health advocacy strategies and will be useful considerations for future public health coalitions in implementing interventions.

**Table 1:** Key lessons from the Victorian Salt Reduction Partnership

	Lessons	Supporting quotes
Step 1. Establishing a sense of urgency	Develop the evidence needed to establish a sense of urgency, such as the economic/business case (e.g. potential for lives saved), and use this to build your coalition, develop your change vision, and generate public and political will	*[Organization] did a scoping study way back in the beginning to identify the evidence and information about successful salt reduction strategies and then provided that to VicHealth about how they could establish a state-wide strategy* (Research 2) *I think that’s a key part of building a business case to government, to show what is feasible* (SGSA 13)
Step 2. Creating the guiding coalition	Invite individuals from different sectors, with different skills and expertise, to create a diverse coalition focused on common goals and capable of developing innovation approaches Beware that this diversity can result in communication challenges and tension regarding public health priorities	*I think those core groups brought a really good mix of skills and expertise, which I think we all learnt from. In my early days on the partnership when we were establishing the agenda, it was really useful and great because we all learned from one another and you know we had some pretty fiery kind of debates and discussions, which were great* (NGO 7) *It’s been really effective to have these different organizations with different skill sets working so closely together* (NGO 21)
Step 3. Develop and maintain influential relationships	Focus on developing and maintaining relationships with policy makers, but also utilize coalition members’ pre-existing relationships Raise the profile of the coalition through media advocacy to facilitate connections with influential people Build relationships with the “opposition” to enable the development of a unified solution to the public health issue	*The partnership has had a positive influence on keeping something happening and helping get it up—certainly useful for [name] as a member of the Healthy Food Partnership to use its work to achieve salt very firmly on the agenda* (NGO 8) *[Name] recommended through the Healthy Food Partnership, as a way of monitoring progress, that those independent surveys be done in exactly the same way* (NGO 8)
Step 4. Develop a change vision	Use a programme logic model to facilitate the development of a comprehensive change vision, including activities, outputs, and outcomes that can be used to monitor programme fidelity and measure success	*Looking at the evidence, engaging with the key stakeholders around it, appraising options for action, feasibility, political acceptability a whole host of different domains to then draw-up a shared plan, on what the consensus for action that everyone could co-commit to* (SGSA 13)
Steps 5 and 6. Communicate the vision for buy-in and empower broad-based action	Identify stakeholders to communicate the change vision to and use active, targeted dissemination strategies to reach them Create and disseminate documents and resources to support communication of the change vision and solutions to the public health issue, including policy position statements and how-to guides Use the media to further communicate your vision	*We developed the call to action document. I think it was a great output and a deliverable… it’s probably the first time organizations have got together to actually get some form of consensus, a blueprint on what we need going forward* (NGO 7) *What’s been really effective has been the product category reports. We used those for strategic advocacy to get media attention* (Research 2)
Step 7. Be opportunistic	Identify potential opportunities to accelerate policy progress and pursue opportunities that are aligned with your change vision to optimize intervention effectiveness	*A reformulation programme was being discussed, being designed, being consulted on, we could then put Partnership responses in* (SGSA 11)
Step 8. Generate short-term wins	Establish approaches to measure or monitor advocacy progress and short-term wins through project outputs that can be linked to outcomes in the programme logic model	*Some good work in terms of lessons from the Victorian Salt Partnership to the Healthy Food Partnership, to offer to share resources, that’s got a good response* (Research 2)
Step 9. Never let up	Be patient and persist while waiting for the right leadership, the right resources, the right time, and the right opportunities	*Like in tobacco control or any other public health area often it can take twenty or thirty years for the advocacy to reach a critical mass and then find a sympathetic minister or a sympathetic government or a sympathetic community and the timing is right and suddenly you get an opportunity* (SGSA 13)
Step 10. Incorporate changes into the culture	Increase public and political awareness through implementing the above steps to change culture and accomplish policy change	*Whilst we might not have seen policy change, we’ve definitely continued the conversation and put support behind it* (NGO 21) *There’s been some good work but as a whole it probably hasn’t quite had the impact at the state and federal level as we would have wanted. That’s not necessarily because of the fault of any of the partners, it’s partly because of the political conversations and agendas out where salt is and you can’t make an issue popular with politicians if they don’t want it to be and there’s not a public push* (SGSA 10)

## Strengths and limitations

This evaluation was guided by a well-established change management framework [18], adapted for public health advocacy strategies [17]. This facilitated the understanding of factors influencing the success of the advocacy strategy, including why and how VSRP advocacy activities and outputs did or did not lead to policy change. However, in using this deductive approach, it is possible that some key factors were not identified as the tool was not used for planning the strategy. As part of the comprehensive process evaluation [19], advocacy activities were documented in real time, ensuring no activities were missed, and supplemented by the semi-structured interviews to gain further insight. Although interviewees’ perspectives may not be representative of all VSRP members, food industry, government, and policy-makers’ perspectives were included. Lastly, additional process evaluation dimensions, such as reach, dose, and adoption, would allow further insight into the success of the strategy, and further evaluation is therefore warranted.

## Conclusions

The Kotter Plus 10-step framework was a useful tool for evaluating the success of the VSRP advocacy strategy for policy change. It allowed reflection on past efforts and enabled assessment of how future efforts might be made more effective. The framework enabled the identification of key strengths, including the creation of the guiding coalition, as well as where advocacy efforts could be improved in future similar strategies, such as effectively communicating the change vision to decision-makers, to better influence policy, and improve public health impact. Use of the Kotter Plus framework as a planning tool, which allows the development of a sequential plan and checklist to ensure the best possible chance of influencing decision-makers, may be a good way of improving advocacy outputs in future coalitions.

## Data Availability

The data sets used and/or analysed during the current study are available from the corresponding author on reasonable request.

## Abbreviations

SGSA: State government or statutory agency
VSRP: Victorian Salt Reduction Partnership

## References

[1] Magnusson RS, Patterson D (2014). The role of law and governance reform in the global response to non-communicable diseases. Glob Health.

[2] Collins T, Mikkelsen B, Adams J, Chestnov O, Evans T, Feigl A (2018). Addressing NCDs: a unifying agenda for sustainable development. Glob Public Health.

[3] Bryan-Jones K, Chapman S (2006). Political dynamics promoting the incremental regulation of secondhand smoke: a case study of New South Wales, Australia. BMC Public Health.

[4] Chapman S (2001). Advocacy in public health: roles and challenges. Int J Epidemiol.

[5] Moore M (2020). Power, politics and persuasion: the critical friend in public health advocacy.

[6] Freeman B, Chapman S, Storey P (2008). Banning smoking in cars carrying children: an analytical history of a public health advocacy campaign. Aust N Z J Public Health.

[7] Chapman S, Wakefield M (2001). Tobacco control advocacy in Australia: reflections on 30 years of progress. Health Educ Behav.

[8] World Health Organization. Global action plan for the prevention and control of noncommunicable diseases 2013–2020. Geneva: World Health Organization; 2013. https://apps.who.int/iris/bitstream/handle/10665/94384/9789241506236_eng.pdf;jsessionid=2E7C16E12B9BA175550D99E7ED59B944?sequence=1.

[9] Moore M, Jones A, Pollard CM, Yeatman H (2019). Development of Australia's front-of-pack interpretative nutrition labelling Health Star Rating system: lessons for public health advocates. Aust N Z J Public Health.

[10] Institute for Health Metrics and Evaluation (IHME). GBD compare data visualization. Seattle: IHME, University of Washington; 2017. https://vizhub.healthdata.org/gbd-compare/.

[11] World Health Organization Regional Office for Europe. European food and nutrition action plan 2015–2020. Copenhagen: WHO Regional Office for Europe; 2015. https://www.euro.who.int/__data/assets/pdf_file/0003/294474/European-Food-Nutrition-Action-Plan-20152020-en.pdf.

[12] Shahid M, Neal B, Jones A (2020). Uptake of Australia’s health star rating system 2014–2019. Nutrients.

[13] Healthy Food Partnership. Healthy food partnership reformulation program: evidence informing the approach, draft targets and modelling outcomes. Canberra: Commonwealth of Australia; 2018. http://www.health.gov.au/internet/main/publishing.nsf/Content/reformulation.

[14] Rosewarne E, Huang L, Farrand C, Coyle D, Pettigrew S, Jones A (2020). Assessing the Healthy Food Partnership’s proposed nutrient reformulation targets for foods and beverages in Australia. Nutrients.

[15] Coyle D, Shahid M, Dunford E, Mhurchu CN, Mckee S, Santos M (2020). Contribution of major food companies and their products to household dietary sodium purchases in Australia. Curr Dev Nutr.

[16] Victorian Health Promotion Foundation (VicHealth). Salt reduction in Victoria. Melbourne: VicHealth; 2014. https://www.vichealth.vic.gov.au/programs-and-projects/salt-reduction.

[17] Moore M, Yeatman H, Pollard C (2013). Evaluating success in public health advocacy strategies. Vietnam J Public Health.

[18] Kotter JP (2012). Leading change.

[19] Trieu K, Jan S, Woodward M, Grimes C, Bolam B, Nowson C (2018). Protocol for the process evaluation of a complex, statewide intervention to reduce salt intake in Victoria, Australia. Nutrients.

[20] Rosewarne E, Chislett W-K, McKenzie B, Trieu K, Webster J (2021). Stakeholder perspectives on the effectiveness of the victorian salt reduction partnership: a qualitative study. BMC Nutrition.

[21] Damschroder LJ, Aron DC, Keith RE, Kirsh SR, Alexander JA, Lowery JCJI (2009). Fostering implementation of health services research findings into practice: a consolidated framework for advancing implementation science. Implement Sci.

[22] Graudal NA, Hubeck-Graudal T, Jürgens G (2012). Effects of low-sodium diet vs. high-sodium diet on blood pressure, renin, aldosterone, catecholamines, cholesterol, and triglyceride (Cochrane Review). Am J Hypertens.

[23] Department of Health. The Victorian Health Monitor Food and Nutrition report. Melbourne: State Government of Victoria; 2012. https://www2.health.vic.gov.au/about/publications/researchandreports/Victorian-Health-Monitor-Food-and-Nutrition-report.

[24] World Health Organization (2007). Prevention of cardiovascular disease: guidelines for assessment and management of cardiovascular risk.

[25] Christoforou A, Trieu K, Land M-A, Bolam B, Webster J (2016). State-level and community-level salt reduction initiatives: a systematic review of global programmes and their impact. J Epidemiol Community Health.

[26] Trieu K, McMahon E, Santos JA, Bauman A, Jolly K-A, Bolam B (2017). Review of behaviour change interventions to reduce population salt intake. Int J Behav Nutr Phys Act.

[27] Victorian Health Promotion Foundation (VicHealth). State of salt: the case for salt reduction in Victoria. Supporting evidence document. Melbourne: VicHealth; 2015. https://www.vichealth.vic.gov.au/-/media/Images/VicHealth/Images-and-Files/MediaResources/Publications/HealthyEating/State-of-Salt/Salt-Reduction-Victoria_supporting-evidence_June15.pdf?la=en&hash=9E257A3E8EB39684E5AFE7DC40BB8D8A05BF5D5B.

[28] Webster J, Trieu K, Dunford E, Nowson C, Jolly K-A, Greenland R (2015). Salt reduction in Australia: from advocacy to action. Cardiovasc Diagn Ther.

[29] Cullerton K, Donnet T, Lee A, Gallegos D (2018). Effective advocacy strategies for influencing government nutrition policy: a conceptual model. Int J Behav Nutr Phys Act.

[30] Rosewarne E, Trieu K, Farrand C, Reimers J, Potter J, Davidson C (2020). Unpack the Salt: an evaluation of the Victorian Salt Reduction Partnership’s media advocacy activities to highlight the salt content of different foods. Nutr J.

[31] Victorian Health Promotion Foundation (VicHealth). Victorians urged to curb their consumption of salt. Melbourne: VicHealth; 2017. https://www.vichealth.vic.gov.au/media-and-resources/media-releases/victorians-urged-to-curb-their-consumption-of-salt.

[32] Allen LN, Feigl AB (2017). What's in a name? A call to reframe non-communicable diseases. Lancet Glob Health.

[33] Frieden TR (2014). Six components necessary for effective public health program implementation. Am J Public Health.

[34] Cullerton K, Donnet T, Lee A, Gallegos D (2016). Playing the policy game: a review of the barriers to and enablers of nutrition policy change. Public Health Nutr.

[35] Butterfoss FD, Kegler M, DiClemente RJ, Crosby RA (2009). The community coalition action theory. Emerging theories in health promotion practice and research.

[36] Willis C, Greene J, Riley B (2017). Understanding and improving multi-sectoral partnerships for chronic disease prevention: blending conceptual and practical insights. Evid Policy.

[37] Taylor-Robinson DC, Lloyd-Williams F, Orton L, Moonan M, O'Flaherty M, Capewell S (2012). Barriers to partnership working in public health: a qualitative study. PLoS ONE.

[38] Willis C, Greene J, Abramowicz A, Riley B (2016). Strengthening the evidence and action on multi-sectoral partnerships in public health: an action research initiative. Chronic Dis Inj Can.

[39] Baker EA, Wilkerson R, Brennan LK (2012). Identifying the role of community partnerships in creating change to support active living. Am J Prev Med.

[40] Victorian Health Promotion Foundation (VicHealth). State of salt: the case for salt reduction in Victoria. Melbourne: VicHealth; 2015. https://www.vichealth.vic.gov.au/-/media/Images/VicHealth/Images-and-Files/MediaResources/Publications/HealthyEating/State-of-Salt/Salt-of-Salt_poster-2015.pdf?la=en&hash=0F2C00980152962361247264CCD6849340B3E97D.

[41] Hawkes C, Buse K (2011). Public health sector and food industry interaction: it’s time to clarify the term ‘partnership’ and be honest about underlying interests. Eur J Public Health.

[42] Cullerton K, Donnet T, Lee A, Gallegos D (2016). Exploring power and influence in nutrition policy in Australia. Obes Rev.

[43] Hunter D, Perkins N (2012). Partnership working in public health: the implications for governance of a systems approach. J Health Serv Res Policy.

[44] Babiak K, Thibault L (2009). Challenges in multiple cross-sector partnerships. Nonprofit Volunt Sect Q.

[45] Peachey JW, Cohen A, Shin N, Fusaro B (2018). Challenges and strategies of building and sustaining inter-organizational partnerships in sport for development and peace. Sport Manage Rev.

[46] Lyn R, Aytur S, Davis TA, Eyler AA, Evenson KR, Chriqui JF (2013). Policy, systems, and environmental approaches for obesity prevention: a framework to inform local and state action. J Public Health Manage Pract JPHMP.

[47] Healthy Food Partnership. Reformulation targets. Canberra: Commonwealth of Australia; 2020. https://www1.health.gov.au/internet/main/publishing.nsf/Content/reformulation-targets.

[48] Trieu K, Webster J. Economic business case for salt reduction action in Victoria. Melbourne: VicHealth; 2015. https://www.vichealth.vic.gov.au/-/media/Images/VicHealth/Images-and-Files/MediaResources/Publications/HealthyEating/State-of-Salt/AppendixC_Salt-Reduction-Vic_Economic-business-case_June15.pdf?la=en&hash=426A475D763EF7B72FD346B7A818E656E6614A92.

[49] Victorian Health Promotion Foundation (VicHealth). Salt partnership update. Melbourne: VicHealth; 2016. https://www.vichealth.vic.gov.au/media-and-resources/blog/salt-partnership-update.

[50] Victorian Salt Reduction Partnership. Reducing the pressure on our health and economy. A call to action from the Victorian Salt Reduction Partnership. Melbourne: National Heart Foundation of Australia; 2018. https://unpackthesalt.com.au/wp-content/uploads/2017/08/HFO0012-Advocacy-Leaflet-A5_V7_LoRes.pdf.

[51] Dodson EA, Eyler AA, Chalifour S, Wintrode CG (2012). A review of obesity-themed policy briefs. Am J Prev Med.

[52] The George Institute for Global Health. Response 296798722. Healthy Food partnership voluntary food reformulation targets—public consultation. Canberra: Australian Government Department of Health; 2018. https://consultations.health.gov.au/population-health-and-sport-division-1/hfp-reformulation/consultation/view_respondent?uuId=296798722.

[53] Victorian Salt Reduction Partnership. Response 1037015099. Healthy food partnership voluntary food reformulation targets—public consultation. Canberra: Australian Government Department of Health; 2018. https://consultations.health.gov.au/population-health-and-sport-division-1/hfp-reformulation/consultation/view_respondent?uuId=1037015099.

[54] Grimes CA, Khokhar D, Bolton KA, Trieu K, Potter J, Davidson C (2020). Salt-related knowledge, attitudes and behaviors (KABs) among victorian adults following 22-months of a consumer awareness campaign. Nutrients.

[55] Victorian Salt Reduction Partnership. Reformulation Readiness. A best practice guide to salt reduction for Australian food manufacturers. Melbourne: Victorian Salt Reduction Partnership; 2019. https://unpackthesalt.com.au/wp-content/uploads/2019/06/Reformulation-Readiness-How-To-Guide.pdf.

[56] Sustainable Development Goals. The victorian salt reduction partnership by The George Institute for Global Health Australia: Global Compact Network Australia; 2020. https://sdgs.org.au/about-us/.

[57] Unpack the Salt. Event summary—3rd May 2019. Melbourne, Australia; 2019.

[58] Healthy Food Partnership. Healthy Food Partnership Communique 13 November 2015. Canberra: Commonwealth of Australia; 2017. https://www1.health.gov.au/internet/main/publishing.nsf/Content/B0653147363CEF33CA257FAD00823950/$File/Healthy%20Food%20Partnership%20Communique%2013%20November%202015.pdf.

[59] Healthy Food Partnership. Work plan for reformulation Working Group (October 2016–December 2017). Canberra: Commonwealth of Australia; 2017. https://www1.health.gov.au/internet/main/publishing.nsf/Content/9BD46D97B65A6209CA257FAD00823957/$File/Reformulation%20workplan.pdf.

[60] Bopp M, Saunders RP, Lattimore D (2013). The tug-of-war: fidelity versus adaptation throughout the health promotion program life cycle. J Primary Prevent.

[61] World Health Organization. Cancer control: knowledge into action: WHO guide for effective programmes. Policy and advocacy. Module 6. Geneva: World Health Organization; 2008. 24716264

[62] Pérez D, Van der Stuyft P, del Carmen ZM, Castro M, Lefèvre P (2015). A modified theoretical framework to assess implementation fidelity of adaptive public health interventions. Implement Sci.

[63] McKenzie B, Trieu K, Grimes CA, Reimers J, Webster J (2019). Understanding barriers and enablers to state action on salt: analysis of stakeholder perceptions of the VicHealth salt reduction partnership. Nutrients.

[64] Public Health Association of Australia (PHAA). National Nutrition Policy. Policy position statement. Canberra: PHAA; 2018. https://www.phaa.net.au/documents/item/3287.

[65] Daube M. McInerney M, editor. Croakey. 2017. https://www.croakey.org/longread-democracy-is-not-a-spectator-sport-11-commandments-for-public-health-advocacy/. Accessed 2020.

[66] Moodie R. Quick Q&A with Rob Moodie on tobacco, obesity, change and leadership. In: McInerney M, editor. Croakey; 2013.

[67] Victorian Health Promotion Foundation (VicHealth). Draft salt targets welcomed for food manufacturers. Melbourne: VicHealth; 2018. https://www.vichealth.vic.gov.au/media-and-resources/media-releases/draft-salt-targets-welcomed-for-food-manufacturers.

